# Coping with Workplace Violence in Healthcare Settings: Social Support and Strategies

**DOI:** 10.3390/ijerph121114429

**Published:** 2015-11-13

**Authors:** Siqi Zhao, He Liu, Hongkun Ma, Mingli Jiao, Ye Li, Yanhua Hao, Yihua Sun, Lijun Gao, Sun Hong, Zheng Kang, Qunhong Wu, Hong Qiao

**Affiliations:** 1 Department of Health. Policy and Hospital Management, School of Public Health, Harbin Medical University, Harbin 150081, China; E-Mails: siqizhao1992@foxmail.com(S.Z.); lhhh314@126.com (H.L.); Liye19832014@126.com (Y.L.); 2 Department of Health Management, Harbin Medical University, Harbin 150081, China; E-Mail:mahongkun222@sohu.com; 3 Institute of Quantitative and Technical Economics, Chinese Academy of Social Science, Beijing 100000, China; 4 Department of Social Medicine, School of Public Health, Harbin Medical University, Harbin 150081, China; E-Mails: hyhyjw@126.com (Y.H.); Lijungao2014@126.com (L.G.); zhengkang1983@126.com (Z.K.); 5 Human Resources Department, Shanghai Mental Health Center, Shanghai 200030, China; E-Mail: sunyihua2014@126.com; 6 Department of Medical Demography, School of Public Health, Harbin Medical University, Harbin 150081, China; E-Mail: sunhong19652014@126; 7 Endocrine and Metabolic Diseases, The 2nd Affiliated Hospital of Harbin Medical University, Harbin 150081, China

**Keywords:** healthcare workers, social support, strategies, workplace violence

## Abstract

A cross-sectional survey of healthcare professionals from 19 hospitals in six cities of Heilongjiang Province, China was conducted. This study had two objectives: (1) to examine the factors influencing healthcare workers’ opinions of strategies to prevent workplace violence, using social support theory, and (2) to encourage healthcare organisations and the larger society to offer greater support to healthcare workers. The respondents exposed to workplace violence expected to receive organisational and social support. Those exposed to psychological violence had a strong opinion of the need for target training to strengthen their competence in responding to violence (OR = 1.319, 95% CI: 1.034–1.658) and enacting workplace violence legislation (OR = 1.968, 95% CI: 1.523–2.543).Those exposed to physical violence thought it might be useful to reinforce staff with back-up support (OR = 3.101, 95% CI: 1.085–8.860). Those exposed to both types of violence and those with high anxiety levels need greater support at both the organisational and societal levels.

## 1. Introduction

The World Health Organization (WHO) categorises workplace violence into two types: (1) physical violence (e.g., beating, kicking, slapping, stabbing, shooting, pushing, biting, and pinching) and (2) psychological violence (e.g., threat of physical force against another person or group that can result in harm to physical, mental, spiritual, moral, or social development) [1]. Workplace violence in the healthcare sector is a worldwide concern, with healthcare workers being at a high risk [2,3]. Workplace violence is common in healthcare settings in China [3,4]. In 2013, a survey conducted by the Chinese Hospital Association found that 27.3 assaults on healthcare workers per hospital were recorded in 2012, which is considerably larger than the number (20.6) recorded in 2008 [5]. These assaults included events in which healthcare workers were killed or seriously injured. In 2012, a patient at the affiliated hospital of Harbin Medical University fatally stabbed an intern who was not involved in his treatment. In late 2013, several news stories shocked China. In Wenling’s First People’s Hospital, an angry patient violently attacked three doctors with a sharp knife, causing the death of one of the doctors and injuries to the others. Two radiologists were beaten by nine people because these individuals were unable to be examined immediately; the assault resulted in the hospitalisation of one of the radiologists [5,6]. Many studies have confirmed that the adverse consequences arising from workplace violence are likely to have effects at the organisational, societal, and individual levels. Individual healthcare workers who have experienced violence show signs of depression, anxiety, low job satisfaction [7], low efficiency in their work performance [8], and a decrease in the quality of the nursing care that they provide [9]. These adverse outcomes may also have direct and indirect costs for the organisation, such as job changes [10] and lower morale [11]. The prevalence of workplace violence may also affect social stability. In 2014, Magnavita found that workers with lower levels of well-being and professional capacity were exposed to violence more frequently than other workers; therefore, the relationship between violence and work stress can be viewed as a chicken-and-egg situation [12].

Some studies have found that social support is negatively related to healthcare workers’ job stress and job dissatisfaction [13,14]. Studies also have reported that social support can buffer the effects of violence on health- and work-related outcomes [15,16]. Therefore, social support may reduce the stress and other adverse consequences experienced by workers who have been exposed to workplace violence.

Mutual support between people presumably has existed as a universal concept since the human race emerged; however, social support as a scientific term was officially introduced in the 1970s. In the early part of that decade, the psychiatric literature introduced the notion of social support. Since then, numerous studies about the relationship between social support and health have been conducted in sociology, and medicine, using quantitative research methods. Researchers have described and defined social support from different perspectives, implying that social support is a multi-dimensional concept. In sociology, social support is viewed as a complex system or network that involves the individual, organisation/community, and society. In a literature review, Hupcey divided the conception of social support into five categories [17] in terms of social networks. Meanwhile, social support may be defined as being accessible to an individual through social ties to other individuals, groups, and the larger community [18]. Regardless of the individual researchers’ perspectives of social support, the most common characteristic of this concept is that it is available through various social relationships that are connected to external resources. Sarason and Kuichen Yang divided social support into three subsystems—individualsupport subsystem, organisational support subsystem, and national support subsystem which constitute the central meaning of social support [19,20]. Social support theory has been most often used in studies of vulnerable groups, such as postpartum depression patients, divorced women with children, and HIV patients [21,22,23]. Some studies have examined healthcare workers as a vulnerable group as well [24,25]; therefore, we decided to examine strategies to prevent and decrease workplace violence using this theoretical framework.

How to prevent and decrease workplace violence is becoming an urgent problem that must be investigated. Some studies have proposed strategies to prevent violence by eliminating risk factors in the work environment [26,27]. A large medical bulletin-board system published a Chinese version of violence prevention guidelines in 2014 for China’s healthcare workers in order to provide authoritative guidelines from a professional perspective. Some studies have recommended training as an important anti-violence strategy, and have examined its protective effects on healthcare workers. Workers who receive violence- prevention training may be more likely to intervene during violent events, whereas their untrained counterparts may be more passive [28]. Training may also improve workers’ problem-solving abilities and coordination skills, helping them predict risk factors for violence and increasing their knowledge of how to avoid situations that are potentially dangerous [29]. Several studies have examined methods to prevent workplace violence specifically among healthcare workers [30,31]. However, in China, few studies exist regarding the strategies to prevent and decrease workplace violence [5]. In this study, we focused on patients and their family members as sources of violence because they comprise the main types of violent incidents [32], and we define workplace violence as a person’s exposure to at least one type of violence (physical or psychological violence).

This study had two objectives: (1) to examine the factors influencing healthcare workers’ opinions of preventative workplace violence strategies using social support theory, and (2) to encourage healthcare organisations and the larger society to offer greater support to healthcare workers by providing empirical evidence for the development of anti-violence policies.

## 2. Materials and Methods

### 2.1. Study Population

A retrospective cross-sectional survey was conducted in Heilongjiang Province, China. Heilongjiang has a population of 38.1 million with 69 tertiary hospitals scattered across 13 cities. Due to the time and resource limitations of this study, six cities (Harbin, Daqing, Qiqihaer,Mudanjiang, Jiamusi, and Jixi) were selected based on the comprehensive rankings of their 2014 Gross Domestic Product (GDPs) and the populations’ health levels. We chose the healthcare workers of these hospitals for our study population because tertiary public hospitals in China have a higher prevalence of workplace violence. Nineteen tertiary public hospitals were recruited for the study and all of them agreed to participate. As most hospitals were located in the largest city—the capital city of Harbin—we selected 12 hospitals there. Two hospitals each were selected from Qiqihaer and Daqing, and one hospital was selected from Mudanjiang, Jiamusi, and Jixi.

### 2.2. Sampling and Data Collection

Cooperation was obtained from the managers and human resource departments of the 19 hospitals, which supplied lists of all their healthcare workers. We combined the lists, numbered the all of the workers’ names, and selected on average 126 personnel from each of the 19 hospitals. The healthcare workers were from various professions, including medicine, nursing, and others (such as management and allied health). After consenting to participate, respondents were asked to complete and return an anonymous questionnaire to a box provided in the manager’s office; respondents’ names and other identifiers were not required. Using this procedure, we obtained 1793 valid questionnaires (total response rate: 89.0%, total valid rate: 83.9%). The data collection period was between 15 July 2014 and 22 October 2014.

### 2.3. Questionnaire

We used a questionnaire developed in 2003 by the International Labor Office (ILO), International Council of Nurses (ICN), WHO, and Public Services International (PSI) joint programto measure workplace violence [33].

First, we obtained permission from the ILO and WHO to use their questionnaire. Then, we asked experts to revise the questions about violence prevention strategies in order to evaluate the instrument’s content validity, including its suitability for use in the Chinese culture and the appropriateness of the translation.These experts included epidemiologists, health management experts, public health specialists, health systems experts, and experts from administrative departments (15 people), who discussed issues regarding the revised questionnaire. We conducted a two-week test-retest reliability check (*r* = 0.87) with a group of 42 healthcare workers from 5 hospitals; the second testing was performed two weeks later. The questionnaire was then back-translated into English to verify the accuracy of the Mandarin version.

The data were collected using the final questionnaire, which consisted of the following four sections:

1. Demographic and workplace data were collected: age, sex, years of experience, and profession. One question measuring anxiety about workplace violence was answered on a scale ranging from 1 (*not at all*) to 5 (*extremely high*).
2. Questions were asked about experiences within the past 12 months, with physical violence (defined as an intentional behaviour that harms healthcare workers physically). Events were measured by asking, “Have you experienced physical violence in the past 12 months”? A “Yes/No” response was required.
3. Questions were asked about experiences within the past 12 months, with psychological violence, including verbal abuse (behaviour that humiliates or degrades a person or a lack of respect for others), threatening events (including offensive behaviour or comments that threaten healthcare workers and that may scare people), and sexual harassment (unwelcome behaviour or comments of a sexual nature that cause people to feel embarrassed or humiliated). Three types of psychological abuse were measured: “How often have you encountered verbal abuse, threatening events, or sexual harassment in the past year?” Participants responded using a scale ranging from 1 (none) to 4 (always).
4. Healthcare workers were asked about choices of effective strategies to prevent workplace violence. This part of the questionnaire consisted of 14 items (strategies), such as using batons, helmets, and other protective equipment, training workers to deal with violence, and enacting workplace violence legislation.

A series of questions assessed the types of support that healthcare workers could receive, and their expectations and evaluations of these sources of support. Such support was divided into three subsystems: individual (4 items: support from co-workers, family, psychologist, and self-support), organisational (4 items: financial compensation, complete accident/injury report, a change of jobs, and reporting to the leader), and social support (4 items: support from police, government, legislation, and social organisations). “Yes/No” responses were required for all of the questions.

### 2.4. Data Analysis

Descriptive statistics were calculated to summarise the demographic characteristics and frequency of healthcare workers’ use of support strategies. Pearson’s chi-square test was used to analyse the differences in exposure to violence according to the respondents’ characteristics. Multiple logistic regression was used to determine which of the respondents’ characteristics predicted their choices of strategies to prevent workplace violence. IBM SPSS Statistics 19.0 was used for the analysis. The level of statistical significance was set as *p* < 0.05.

### 2.5. Ethics Statement

The administrators of the 19 hospitals approved the research protocol, including the purpose, method, and use of the data collected. All participants received a notification letter, which consisted of the survey instrument, cover letter, and consent form describing the study goals and issues of confidentiality. All participants provided informed consent in writing along with their completed questionnaires. The study protocol was reviewed and approved by the Research Ethics Committee of Harbin Medical University.

## 3. Results

### 3.1. Demographic Characteristics of the Respondents

Of the 1793 healthcare professionals who responded to the questionnaire, 170 (9.5%) reported that they had been exposed to physical violence during the previous 12 months. and 1241 (69.2%) reported that they had been exposed to psychological violence during the same period. The characteristics of the healthcare workers who were exposed to violence also are presented in Table 1. Exposure to physical violence significantly differed by sex, years of experience, department, shift work, and anxiety level. Exposure to psychological violence significantly differed by sex, age, years of experience, profession, shift work, and anxiety level.

**Table 1:** Frequency distributions of the characteristics of the workers exposed to physical and psychological violence.

Characteristics		Physical				Psychological			
		N ^a^	%	χ^2^	*p*	N ^b^	%	χ^2^	*p*
Gender	Males	92	13.4%	19.842	<0.01	500	73.5%	6.015	0.014
	Female	78	7.1%			741	68.0%		
Age	≤25	19	8.7%	4.168	0.244	167	77.0%	21.07	<0.01
	26–35	75	8.9%			609	72.4%		
	36–45	57	11.7%			326	68.5%		
	>45	19	7.8%			139	59.1%		
Years of experience	≤5	39	8.0%	10.906	0.028	344	71.1%	31.05	<0.01
	6–10	46	9.7%			342	72.8%		
	11–15	34	13.5%			188	96.4%		
	16–20	22	12.8%			131	76.2%		
	>20	29	7.1%			236	59.4%		
Profession	Physicians	104	10.8%	4.669	0.198	701	73.6%	41.24	<0.01
	Nurses	46	7.8%			417	71.4%		
	Medical Technicians	6	7.1%			49	58.3%		
	Others	14	8.9%			74	49.7%		
Department	Medical	54	7.8%	11.924	0.018	486	70.8%	6.469	0.167
	Surgical	72	10.4%			511	74.4%		
	Medical Technicians	4	7.3%			35	63.6%		
	Emergency	10	22.2%			37	82.2%		
	Other	10	9.8%			74	72.5%		
Work in shifts	Yes	140	11.1%	12.905	<0.01	952	75.9%	67.11	<0.01
	No	30	5.6%			289	56.2%		
Anxiety level	Extremely high	120	17.5%	83.508	<0.01	521	76.4%	80.17	<0.01
	High	13	5.0%			194	76.4%		
	Moderate	26	4.8%			379	70.6%		
	Low	6	3.4%			95	53.7%		
	Zero	5	4.1%			52	43.7%		

Notes: N ^a^ = 170 (people exposed to physical violence); N ^b^ = 1241 (people exposed to psychological violence).

### 3.2. The Support Strategies Healthcare Workers Used and Their Expectations

Table 2 shows the types of help that healthcare workers sought after they were exposed to violence. We classified the types of support received as the individual support subsystem and organisational support subsystem. When they were exposed to physical violence, the highest proportion of respondents (66.7%) reported using self-defence, followed by talking with co-workers (31.5%), which were categorised as individual support. The organisational support subsystem included assistance or programs that the organisation made available to the healthcare workers. The highest proportion of respondents (29.4%) received support from their organisation by reporting workplace violence in a timely manner (*i.e.*, report to the leader), followed by financial compensation (3.6%). When they were exposed to psychological violence, the highest proportion of respondents (43.6%) received individual support by talking with co-workers, followed by receiving comfort from family members (27.5%). The highest proportion of respondents (27.6%) received organisational support by reporting to the leader, followed by completing an accident/injury report (1.5%).

One quarter (25%) of the workers accepted training for violence prevention, and 80% of respondents were willing to accept training. The organisation can provide these support measures to healthcare workers to deal with violent events. Most respondents who were exposed to violence (physical and psychological) reported being very dissatisfied (63.9% and 54.6% respectively) when they evaluated the level of organisational support overall.

**Table 2:** Methods of obtaining support after exposure to violence.

Support	Ways to Obtain Support after Exposure to Physical ^a^ (Psychological ^b^) Violence	*N* ^a^ (%)	*N* ^b^ (%)
Individual	Self-defence	110(66.7%)	292(25.0%)
	Talk with co-workers	52(31.5%)	509(43.6%)
	Get support from family	31(18.8%)	321(27.5%)
	Get support from psychologist	5(2.9%)	21(1.8%)
Organisational	Financial compensation	6(3.6%)	8(0.7%)
	Complete accident/injury report	2(1.2%)	18(1.5%)
	Change jobs	3(1.8%)	14(1.2%)
	Report to leader	50(29.4%)	322(27.6%)

Notes: ^a^ people exposed to physical violence; ^b^ people exposed to psychological violence.

Table 3 shows the support subsystems that healthcare workers identified as the subsystems they expected to use if they were exposed to violence. The options are divided into three support subsystems (individual, organisational, and social). The individual and organisational support subsystems have been discussed in the previous section. In addition, the social support subsystem included obtaining help through police, government, legislation, and social organisations, such as the Medical Association and the Staff Safety Association. The highest proportion of respondents who were exposed to physical violence expected to use the organisational support subsystem (*n* = 73, 42.9%), followed by the social support subsystem (*n* = 85, 50.0%); their individual support subsystem was ranked lowest (*n* = 12, 7.1%). The workers who were exposed to psychological violence ranked the subsystems in the same order. Given these healthcare workers’ opinions of the status of support, organisations and society should provide sufficient levels of support to them.

**Table 3:** Healthcare workers’ expected use of support subsystems.

Support Subsystem	*N* ^a^ (%)	*N* ^b^ (%)
Individual support subsystem	12 (7.1%)	170 (13.7%)
Organisational support subsystem	73 (42.9%)	657 (52.9%)
Social support subsystem	85 (50.0%)	414 (33.4%)

Notes: ^a^ people exposed to physical violence; ^b^ people exposed to psychological violence.

### 3.3. Multiple Logistic Regression of Healthcare Workers’ Opinions of Violence Prevention Strategies

We used the respondents’ general characteristics and their experiences of exposure to violence in the multiple logistic regression analysis to examine the factors influencing their choices of the most useful strategies to prevent workplace violence. We divided the anti-violence measures into individual, organisational, and social levels, based on their sources of support. As shown in Table 4, the following strategies—target training, improving communication skills, and improving competence in diagnosis and treatment—had the same factor in common: shift work. These workers thought that these three strategies were the least useful. Two strategies *i.e.*, target training and enacting workplace violence legislation, had one factor in common: exposure to psychological violence. Meanwhile, those who were exposed to physical violence thought that it would be useful to reinforce staff with back-up support. Those exposed to both types of violence evaluated the following strategies as effective methods: the use of protective equipment; hospital improvements in violence reporting, statistics, and interventions; security patrols; reinforcing staff with back-up support; having police officers stationed in the hospital; and the enactment of workplace violence legislation. Healthcare workers’ anxiety levels might have affected their choice of the following strategies: target training; hospital improvements in violence reporting, statistics, and interventions; security patrols; reinforcing staff with back-up support; and promoting transparency of fees. All of these strategies had strong associations with workers with high anxiety levels, and at the social level, they were affected by this factor.

**Table 4:** Multiple logistic regression of the factors influencing workers’ choices of strategies to prevent workplace violence.

Dimensions	Strategies	Variables	*OR*	95% CI	*p*
Individual	Use batons, helmets, and other protective equipment	Profession			
		Nurses	-	-	-
		Medical Technicians	0.428	0.221–0.830	0.012
		Others	-	-	-
		Violence			
		physical violence only	-	-	-
		psychological violence only	-	-	-
		both types of violence	2.026	1.356–3.027	<0.001
	Improve competence in diagnosis and treatment	Working rotating shifts	0.778	0.609–0.993	0.044
		Violence			
		physical violence only	-	-	-
		psychological violence only	0.654	0.518–0.825	<0.001
	Improve doctor-patient communication skills	Working rotating shifts	0.703	0.554–0.891	0.004
		Violence			
		physical violence only	-	-	-
		psychological violence only	0.723	0.567–0.922	0.009
		both types of violence	-	-	-
Organisational	Target training to strengthen competence in responding to violence	Age	0.867	0.768–0.980	0.022
		Working rotating shifts	0.642	0.499–0.826	0.001
		Anxiety level	1.208	1.111–1.313	<0.001
		Profession			
		Nurses	-	-	-
		Medical Technicians	1.709	1.035–2.820	0.036
		Others	-	-	-
		Violence			
		physical violence only	-	-	-
		psychological violence only	1.309	1.034–1.658	0.025
		both types of violence	-	-	-
	Hospital improvements in violence reporting, statistics, and interventions	Anxiety level	1.152	1.054–1.258	0.002
		Violence			
		physical violence only	-	-	-
		psychological violence only	-	-	-
		both types of violence	1.848	1.194–2.860	0.006
	Security patrols in the emergency department and other key departments	Working rotating shifts	0.688	0.523–0.906	0.008
		Anxiety level	1.203	1.100–1.316	<0.001
		Violence			
		physical violence only	-	-	-
		psychological violence only	-	-	-
		both types of violence	1.686	1.058–2.688	0.028
	Reinforce staff with back-up support	Anxiety level	1.208	1.102–1.326	<0.001
		Profession			
		Nurses	1.664	1.307–2.119	<0.001
		Medical Technicians	-	-	-
		Others	1.54	1.029–2.307	0.036
		Violence			
		physical violence only	3.101	1.085–8.860	0.035
		psychological violence only	-	-	-
		both types of violence	1.508	1.003–2.267	0.049
	Improve the treatment process and shorten the waiting time	Violence			
		physical violence only	-	-	-
		psychological violence only	0.693	0.551–0.870	0.002
		both types of violence	-	-	-
	Install cameras on wards, keep work areas bright by using lights at night	Profession			
		Nurses	1.256	1.002–1.574	0.048
		Medical Technicians	-	-	-
		Others	-	-	-
	Promote transparency of fees, assure payment of fees	Anxiety level	1.127	1.030–1.233	0.01
Social	Police officers stationed in the hospital	Sex	0.667	0.501–0.889	0.006
		Anxiety level	1.34	1.217–1.476	<0.001
		Profession			
		Nurses	1.4	1.059–1.851	0.018
		Medical Technicians	-	-	-
		Others	-	-	-
		Violence			
		physical violence only	-	-	-
		psychological violence only	-	-	-
		both types of violence	2.155	1.401–3.317	<0.001
	Enact workplace violence legislation	Age	1.209	1.061–1.379	0.004
		Anxiety level	1.213	1.106–1.331	<0.001
		Department			
		Internal	-	-	-
		Medical Technology	-	-	-
		Emergency	-	-	-
		Others	1.824	1.110–2.999	0.178
	Enact workplace violence legislation	Violence			
		physical violence only	-	-	-
		psychological violence only	1.968	1.523–2.543	<0.001
		both types of violence	2.113	1.344–3.323	0.001
	Develop violence prevention guidelines and plans	Anxiety level	1.136	1.0418–1.239	0.004
		Department			
		Internal	-	-	-
		Medical Technology	0.465	0.247–0.876	0.018
		Emergency	-	-	-
		Others	-	-	-
	Correct perspective and reports by media, promote respect of medical workers	Anxiety level	1.25	1.141–1.368	<0.001

## 4. Discussion

### 4.1. The Importance of Organisational and Social Support

Our findings indicated that healthcare workers received little support from the organisations in which they worked after exposure to workplace violence. Their sources of support were themselves, family, friends, and co-workers with whom they had the closest relationships. However, they expected more support from the organisation and the larger society. Because the organisation is influenced by the broader social environment, its own social atmosphere and its support of healthcare workers might also be affected likewise. The social organisation has the potential to increase an individual’s perception of support in the social environment, thereby making the individual feel that he or she is cared for and valued.

Additionally, several studies have found that vulnerable groups, such as healthcare workers and patients, need organisational and social support [16]. Thus, it is not difficult to understand healthcare workers’ need for more assistance from the organisation and society. If they cannot obtain sufficient support after exposure to violence, a decline in their quality of work and other adverse consequences can be expected. Previous studies have shown that higher levels of organisational support for those who have experienced violence are effective in reducing their tension and stress [14]. A trusted and just work environment may reduce violence against nurses [34]. Therefore, we should increase organisational support to healthcare workers who have been exposed to violence by providing psychological care, supporting their efforts to claim compensation, establishing and improving violence-reporting systems, and training them to improve their competence in responding to violence.

Society’s attitude toward workplace violence should be one of zero tolerance, such that violent behaviour is regarded as a crime that is unacceptable and punishable by law [35]. Currently, there are many countries and regions that have implemented a zero-tolerance policy, which is designed to encourage healthcare workers to report violent incidents and to increase the severity of punishment for it. In 1999, the United Kingdom’s Department of Health, as part of its “zero-tolerance zone” initiative, directed the National Health Service to reduce incidents of violence by 30% by 2003 [36]. Australia’s largest professional and industrial organisation, the Australian Nursing Federation, endorsed a zero-tolerance response to aggression [37]. In addition, research institutions should provide anti-violence workplace guidelines to healthcare workers to help employees increase their awareness of violence and violence prevention skills. No professional institution in China has established authoritative or systematic guidelines or programs to prevent workplace violence; yet the National Institute for Occupational Safety and Health and the Occupational Safety and Health Administration state that healthcare organisations have a duty to provide a safe environment for their employees [31,38].

### 4.2. Workers’ Choices of Useful Strategies to Prevent Violence

In our study, healthcare workers revealed the anti-violence strategies that they deemed useful from the individual, organisational, and social levels of prevention and support. The results of our multivariate logistic regression indicate that different people have different requirements for prevention of workplace violence strategies.

Shift work appeared to be an influential background factor that affected workers’ opinions about prevention strategies. Shift workers identified the following strategies as being significantly less useful: communication skills, improvement of competence in diagnosis and treatment, and target training to strengthen organisational competence in responding to violence. Previous studies have shown that working rotating shifts is a risk factor for violence and there is evidence to support the notion that improving personal competence and skills (through training, for example) might have a protective role in preventing workplace violence [3,28,39,40]. However, shift workers in this survey did not regard these strategies as effective ways to prevent violence on a personal level, which might reflect their opinion that preventing workplace violence cannot occur at this level because shift work is a risk factor for workplace violence.

Regarding violent situations, those exposed to psychological violence had strong opinions about target training to strengthen competence in responding to violence and enacting workplace violence legislation. This study revealed that these healthcare workers had a strong desire and need for training, but the actual number of training sessions that the organisations provided was very low, indicating that insufficient support was provided to the workers. The present study confirmed that training is an important component in the successful prevention of workplace violence [28,29]. Most experts in the field of workplace violence consider a typical training course as one that addresses theory (understanding workplace violence, types of violence, and characteristics of violence) and prevention (assessment of danger and taking precautions) [41,42,43]. Recently, increasing numbers of researchers and healthcare workers have advocated for legislation to protect workers’ physical security [44,45].

In our study, those exposed to psychological violence thought that enacting legislation was necessary. In general, physical violence accompanies abuse and threats, which may be why people look for protection from the law. Although the medical profession has legal oversight and workplace operations have regulations, there are no specific laws to protect basic personal safety, especially in China’s healthcare system. Therefore, special legislation for healthcare workers is necessary to protect their safety. In the United States, California, Colorado, Iowa, Illinois, Pennsylvania, South Carolina, Massachusetts, and New York have enacted legislation to prevent workplace violence and increase the penalties for assaults against nurses [46]. Enacting such legislation might help to decrease workplace violence, as it is designed to protect healthcare workers’ legitimate rights and interests and can be a warning to potential criminals.

Those exposed to physical violence viewed reinforcing staff with back-up support as a potentially useful strategy. Previous studies have confirmed that a hospital is an environment with a heavy workload where a shortage of staff is a potential risk factor for workplace violence [47]. Staff shortages may lead to assaults on staff, as patients or their family may believe that they did not receive timely or high-quality services. It may even cause medical errors that can more easily result in conflict between staff and patients [48].

Those exposed to both types of violence seem to regard the following strategies as effective means of prevention: using protective equipment; improving hospitals’ violence reporting, statistics, and interventions; using security patrols; reinforce staff with back-up support; assigning police officers in the hospital; and enacting workplace violence legislation. These strategies belong to the three subsystems of social support. Healthcare workers who have experienced physical and psychological violence may have thought that preventing workplace violence required a combination of individual, organisational, and societal efforts; their choices seemed more comprehensive compared to the other groups. However, it is obvious that at the personal level, the use batons, helmets, and other protective equipment may not be a permanent solution. The nature of violence is such that it often is instantaneous, and healthcare workers must rely on their own strength to protect themselves until help arrives. Because of fears for their own safety, the healthcare workers in our study wanted to be equipped with protective equipment, which is understandable. This manner of dealing with violence is likely to increase anxiety in healthcare workers, and carrying inconvenient equipment may affect the quality of healthcare services. Therefore, organisations and society should provide more support to these individuals.

Our findings indicate that anxiety levels might have affected healthcare workers’ choices of strategies. At the organisational level, target training, hospitals’ improvements in violence reporting, statistics, and interventions, security patrols, reinforcing staff with back-up workers, and promoting transparency of fees had strong associations with workers with high anxiety levels. At the societal level, all of the strategies have received their approval. We may conclude that workers with high anxiety levels may have greater expectations of organisations and society, which may provide support to relieve their anxiety.

Due to the time and resource limitations of this study, we chose only 19 hospitals, which limit our ability to generalise the study’s findings to all healthcare workers in the Heilongjiang Province. The retrospective reporting of events and activities during the past year may have affected the accuracy of the results. However, our research of the status of prevention strategies from the perspective of social support theory may be valuable for controlling workplace violence and increasing the awareness of the public and policymakers about the need to provide useful support to healthcare workers.

## 5. Conclusions

The results of this study should assist in the development of appropriate policies and strategies to deal with workplace violence against healthcare workers. These strategies should be implemented for specific populations, as each of them have different requirements.

Healthcare workers who have been exposed to both physical and psychological violence hope for protection from further suffering through the enactment of legislation. For those who have been exposed to psychological violence, target training may be useful to prevent violence. For those who have been exposed to physical violence, organisations should reinforce staff with back-up support. For those who have been exposed to both types of violence, organisations should improve their violence reporting systems, statistics, and interventions. Security forces in key departments should be strengthened. The last four strategies also can be used to relieve healthcare workers who have high anxiety levels.

Organisations and society should provide additional support to prevent workplace violence. In a future study, we will examine the utility of these strategies in decreasing workplace violence.
